# Evaluation and Prediction of Mass Transport Properties for Porous Implant with Different Unit Cells: A Numerical Study

**DOI:** 10.1155/2019/3610785

**Published:** 2019-04-23

**Authors:** Jian Li, Diansheng Chen, Yubo Fan

**Affiliations:** ^1^Robotic Institute, Beihang University, Beijing 100191, China; ^2^Beijing Key Laboratory of Rehabilitation Technical Aids for Old-Age Disability and Key Laboratory of Rehabilitation Aids Technology and System of The Ministry of Civil Affairs, National Research Center for Rehabilitation Technical Aids, Beijing 100176, China; ^3^Beijing Advanced Innovation Center for Biomedical Engineering, Beihang University, Beijing 100191, China; ^4^Key Laboratory for Biomechanics and Mechanobiology of Ministry of Education, School of Biological Science and Medical Engineering, Beihang University, Beijing 100191, China

## Abstract

Efficient exchange of nutrients and wastes required for cell proliferation and differentiation plays a pivotal role in improving the service life of porous implants. In this study, mass transport properties for porous implant with different unit cells were evaluated and predicted when the porosities are kept the same. To this end, three typical unit cells (diamond (DO), rhombic dodecahedron (RD), and octet truss (OT)) were selected, in which DO displayed diagonal-symmetrical shape, while RD and OT share midline-symmetrical structure. Then, single unit cells were designed quantitatively, and its shape parameters were measured and calculated. Moreover, corresponding porous scaffolds with same outline size were created, respectively. Furthermore, using computational fluid dynamics (CFD) methodology, flow performances with Dulbecco's Modified Eagle's Medium (DMEM) in vitro were simulated for three different porous implants, and flow trajectory, velocity, and wall shear stress which could reflect the properties of mass transfer and tissue regeneration were compared and predicted numerically. Results demonstrated that different unit cell could directly lead to different mass transport properties for porous implant, in spite of same porosity, scaffold size, and service environment. Additionally, by the results, DO displayed greater tortuosity, more appropriate areas, and smoother shear stress distribution than RD and OT, which would provide better surroundings for implant fixation and tissue regeneration. However, RD and OT showed better mass transport properties because of bigger maximum velocity (5.177 mm/s, 4.381 mm/s) than DO (3.941 mm/s). This study would provide great helps for unit cell selection and biological performance optimization for 3D printed bone implants.

## 1. Introduction

It is well known that an ideal bone implant should match the mechanical properties of the host bone, but, importantly, efficient exchange of nutrients and wastes required for cell proliferation and differentiation also plays a pivotal role in improving the service life of bone implants [1]. To this end, many conventional fabrication techniques, such as gas foaming, solvent casting, particle leaching, fiber meshes, and freeze drying [2], were used to create porous structure to lower the strength and promote cell proliferation and differentiation. Herein, porosity, as an important parameter for porous structure [3], attracted a lot of attentions in the recent years. For porous implant, porosity not only plays a critical role in the mechanical properties [4–6] but also affects biological performances, such as cell attachment, proliferation, and differentiation [7], and transport of nutrients and metabolic waste [8]. In contrast to the solid metal implant, conventional fabrication techniques could provide porous implant with different porosity and low strength. It is, however, that the pore distribution usually has random features for porous implant manufactured by conventional fabrication techniques, and its connectivity may not keep the same and control accurately. Additive manufacturing (AM), also, namely, 3D printing or rapid prototyping, is a process of join materials layer by layer, which provides required ability to deliver a high level of control over the complex architecture of the construct [9]. With the advent of additive manufacturing (AM), many applications benefit from it. Particularly, 3D printed porous implant is one of the medical applications, which has been widely acknowledged as an important and hopeful future development for bone tissue engineering because of personalized customization and controllability [10]. In this regard, many studies had been involved and performed recently [11], and also some patient-specific implants were designed and printed [12, 13], although the abovementioned studies confirmed 3D porous implants have lighter weight, lower stiffness, and a controlled structure [10], which could be optimized and tailored to avoid stress shielding and promote mass transfer, cell adhesion, and differentiation effectively [14]. However, different pore shapes and porosity usually have completely different mass transport properties. As mentioned before, this would lead to different regeneration efficiency and service efficiency for the tissue and implant. To some extent, some improper porous structure creation may be detrimental to cell proliferation and tissue regeneration, thereby affecting implant fixation and long-term service. Meanwhile, ideally, porous implant design is a key step to ‘fit' the implant with appropriate mechanical properties and biological performance in a typical design [15]. However, currently in literature, most of the different kinds of pores are selected just by engineers' experience, lacking quantitative assessment and prediction. In particular far less attention has been paid to mass transport properties. In addition, although literatures had studied different kinds of pores [16]; to date, a significant amount of work has not focused on different symmetric types with same porosity together for the porous structure's mass transport performances in the application of bone implant.

Accordingly, for the evaluation of biological performances of bone implant, methodology in vivo and in vitro could be divided into four kinds: (1) cell culture in vitro; (2) animal experiment in vivo; (3) clinical trial in vivo; (4) numerical analysis in vitro. In general, cell culture, animal experiment, and clinical trial often need long period and lots of money as well as many times' repetition. In the meantime, the results of above methods could be affected by many factors. Relatively, numerical analysis with computational fluid dynamics (CFD) provides potential assistance for bone implant because of quick speed, low cost, and good anti-interference performance. In the aspect of blood vessels, CFD is a reliable and fast method for the flow fluid evaluation, and the validity was confirmed by many researchers [17, 18]. Furthermore, recently, lots of previous studies had evaluated the biological properties of porous implant with CFD method [19–22] and found many useful and amazing results.

Therefore, based on the above state and our previous study [23], this study focused on the regular unit cell and chose one diagonal-symmetrical regular unit cell and two midline-symmetrical regular unit cells, which are commonly used in actual design. From the biological viewpoints, singe unit cells were design quantitatively, and corresponding scaffolds were created with same porosity and scaffold size. Using CFD method, flow trajectory, flow velocity, and flow shear stress of different porous scaffolds in Dulbecco's modified Eagle's medium (DMEM) were predicted for mass transport properties numerically.

## 2. Methods

### 2.1. Pore Preparation and Scaffold Design

**Figure 1:**
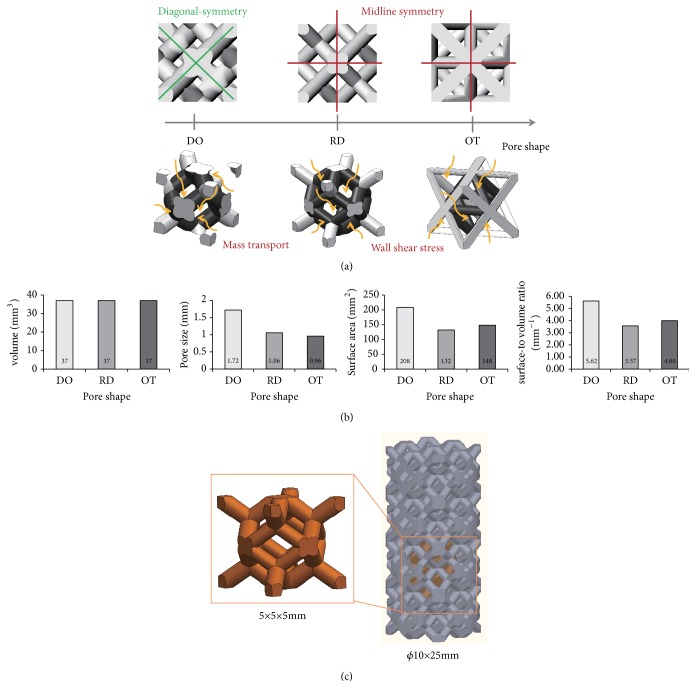
Different unit cells and related porous scaffold: (a) DO, RD, and OT shape; (b) physical parameters of three shapes; (c) schematic diagram of porous structure building. Importantly, the midline-symmetry and diagonal-symmetry described herein are relative to the cube outline.

As shown in Figure 1(a), three unit cells (Diamond (DO) [24], rhombic dodecahedron (RD) [25], and Octet truss (OT) [26]) were selected in this study, which are commonly used in porous implant design, and RD and OT share same midline-symmetrical structure both on the coronal plane and the sagittal plane, while DO displays diagonal-symmetrical shape. Furthermore, porosity as an important parameter for porous implant and its biological properties was kept same (70%) when the three shapes of unit cells (5×5×5mm, Figure 1(a)) were designed in the commercial 3D-design software of SolidWorks (Dassault Systems, Velizy-Villacoublay, France). Accordingly, taking into account quantitative assessment, factors affecting the biological performance were paid more attentions, and unit cell's volume, max-pore size, surface area, and surface-to volume ratio were recorded, computed and compared in Figure 1(b). Specifically, volume and surface area were analyzed automatically in the SolidWorks software, and max-pore size could be measured manually. Further on, porosity and surface-to volume ratio were calculated as [23, 27]

(1) p=V0−VV0×100%

(2) s=VS∗

where *V*_0_ and *V* are the volumes of the solid initial structure and porous structure, respectively, and *S*^*∗*^ is the surface area of porous structure.

With regard to porosity, because DO, RD, and OT were recorded as same volume and unit cells size (5×5×5mm), the same porosity was tested by (1). It is, however, that max-pore size, surface area, and surface-to volume ratio are quite different from each other in spite of sharing same volume and porosity, which could be indicated different biological performance and mechanical properties in the future.

In the case of same porosity (70%) and equal unit cells size (5×5×5mm), single unit cells were repeated along X, Y, and Z axis periodically. Then, three kinds of porous scaffolds with the size of 10 mm in diameter and 25 mm in height were constructed by Boolean operations (Figure 1(c)), respectively.

### 2.2. Mass Transport Properties Prediction

**Figure 2:**
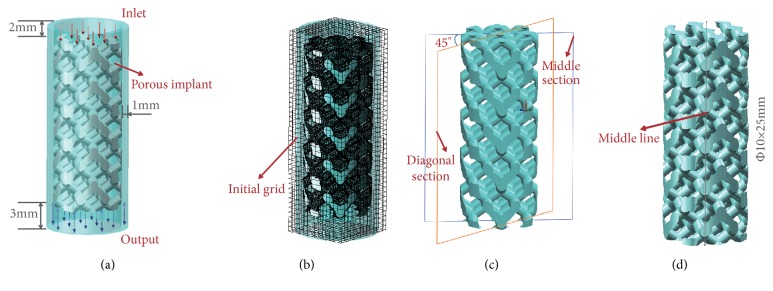
Schematic diagram of CFD model: (a) computational domain; (b) Initial grids and meshing; (c) diagonal section and middle section views; (d) middle line view of the porous scaffold.

**Figure 3:**
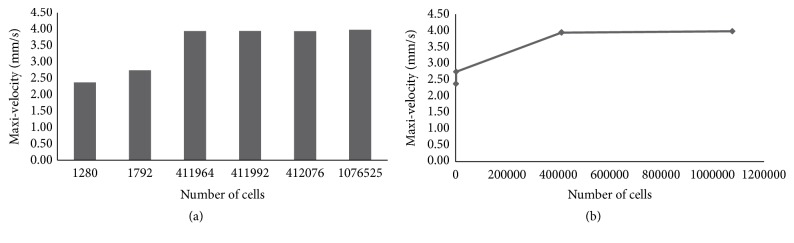
Convergence analysis of CFD model: (a) maximum velocity of the same model with different mesh sizes; (b) change trend of the values of maximum velocity for same model.

Learn from literatures [19–22] that in order to predict the mass transport properties of different porous implants with midline-symmetrical and diagonal-symmetrical unit cells, CFD methodologies were used in this study. Moreover, in the SolidWorks software, corresponding CFD plugin was used to simulate the porous scaffolds in vitro, and flow trajectory, velocity, and wall shear stress which could reflect the properties of mass transport and tissue regeneration were studied. As illustrated in Figure 2, porous models (Figure 2(a), Φ10×25mm) were limited in an enclosed tube (Figure 2(a)), respectively, and one side of the tube was assumed as the inlet, whilst the opposite side was assumed as the outlet. In this study, DMEM commonly used in cell culture was represented as fluid material (incompressible and continuous Newtonian fluid) to simulate a steady state in vitro, whose viscosity and density are 1.45Pa·s and 1000 kg/m^3^ [28]. Namely, the enclosed tube was filled with DMEM, and then porous scaffolds were immersed in one by one. Meanwhile, for the three shapes, the same boundary conditions were defined as: an inlet velocity (v_i_ = 1 mm/s) at the inlet-flow side and an output environmental pressure (one Atm pressure) at the opposite-flow side [23]. It is worth noting that the aim of the chosen boundary conditions was to imitate cell culture of porous implant in vitro. Besides, diagonal section, middle section (Figure 2(c)), and middle line views (Figure 2(d)) of the porous scaffolds were stressed and used to display inner flow velocity and shear stress [23]. Furthermore, the governing equation underlying the calculation was the Navier-Stokes formulation in this study, which could be expressed as (3). Conventionally, Navier-Stokes usually describes the motion of viscous fluid substances and reflects the basic mechanical law of viscous fluid flow, which has great significance in fluid mechanics.

(3) $$\begin{array}{c} \frac{\partial v}{\partial t}+\left(\;v∙\nabla \;\right)v=-\frac{1}{\rho }\nabla p+\frac{\mu }{\rho }\nabla^{2}v \end{array}$$

where *v* is the flow velocity, which varies with the time; *ρ* and *μ* denote the density and dynamical viscosity of DMEM, respectively, which usually have constant value;* p* is the pressure. Accordingly, tetrahedron was used for the models' meshing (Figure 2(b)) in the flow simulation, and adaptive optimization is performed in the software. Moreover, in order to provide accurate computation and reliable results, convergence studies with different initial grids (Figure 2(b)) and mesh sizes (Figure 3(a)) were also conducted to evaluate mesh size as well as calculating time. Meanwhile, change trend of the values of maximum velocity for same model was performed (Figure 3(b)). As Table 1 shown, more than 380000 tetrahedral elements and 260 iterations were included in the three classes of simulations, respectively, in order to bring credible solutions.

## 3. Results 

### 3.1. Flow Trajectory and Velocity

**Figure 4:**
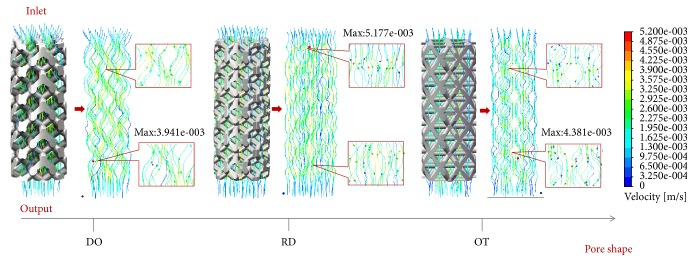
Flow trajectory and velocity distributions of the three porous scaffolds.

**Figure 5:**
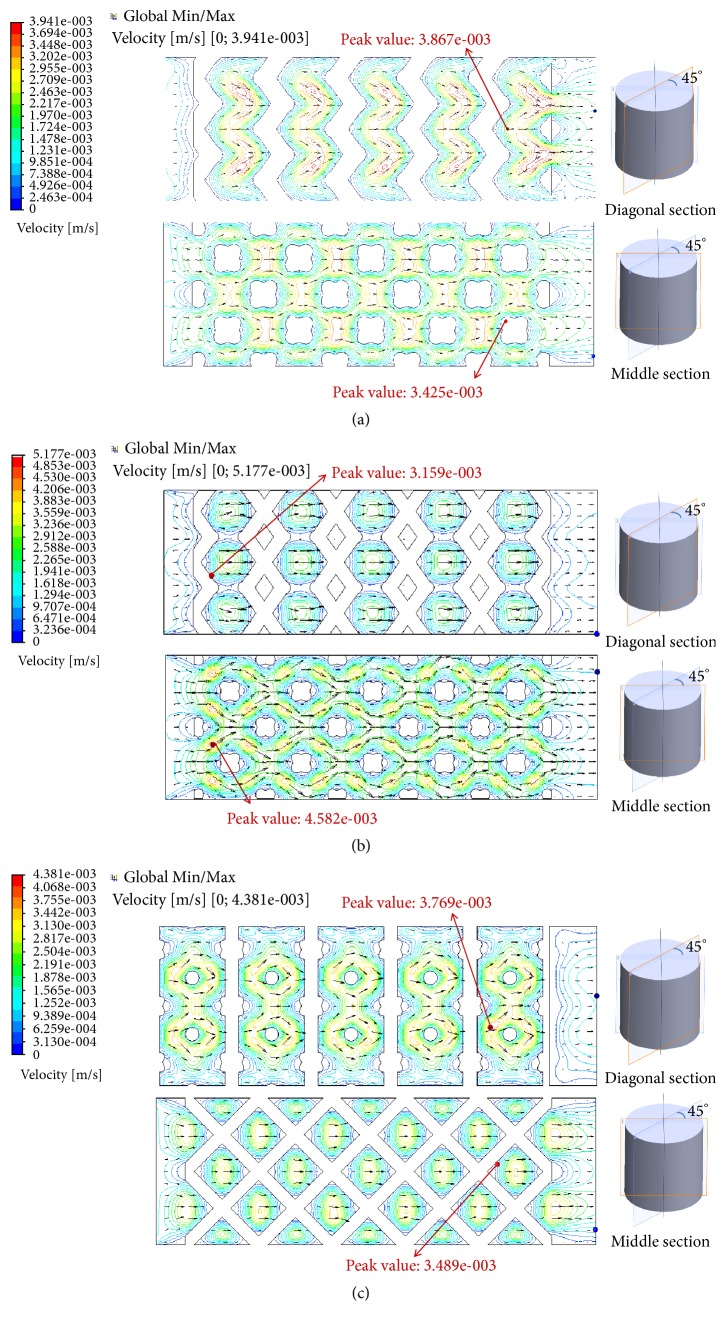
Flow velocity distributions on the diagonal section and middle section: (a) DO; (b) RD; (c) OT.

**Figure 6:**
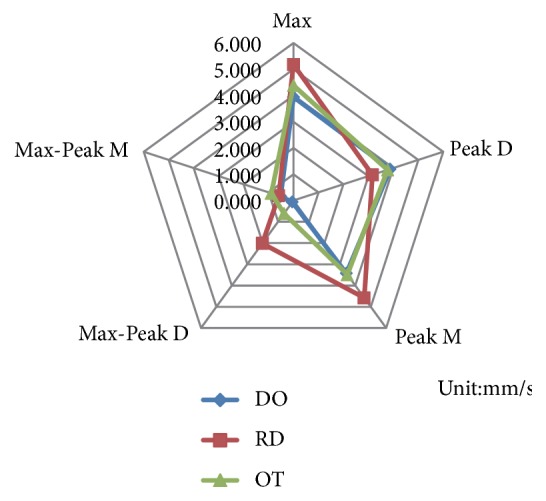
Data analysis of maximum flow velocity in the global view, diagonal section, and middle section.

**Figure 7:**
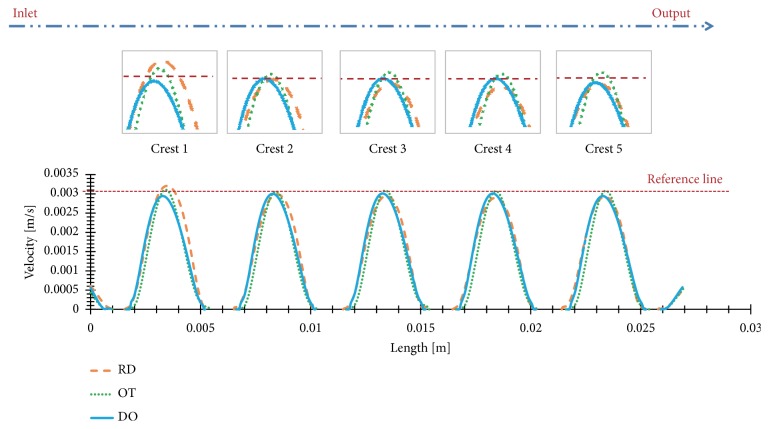
Flow velocity distributions of the three porous scaffolds along the middle line.

As illustrated in Figure 4, flow trajectory and velocity distributions of the three porous scaffolds (DO, RD, and OT) with the same porosity are elaborated in the global view, in which maximum velocity values observed in were 3.941 mm/s, 5.177 mm/s, and 4.381 mm/s, respectively. Besides, in order to compare the results intuitively, the same numbers of trajectory lines were depicted and amplified by the uniform multiple. In the meantime, one cloud chart was shared for the three shapes [23]. Obviously, curvatures of the trajectories were quite different from each other, and the descending order of flow velocity is RD, OT, and DO. In addition, for an easy understanding of the inner flow velocities, flow velocity distributions on the diagonal section and middle section could be found in Figure 5, and corresponding data analysis of maximum velocity values in global view, diagonal section, and middle section were was directly in Figure 6. Furthermore, in Figure 7, flow velocities along the middle line were illustrated and compared for the three shapes. As expected, above details also showed quite difference for DO, RD, and OT shapes, such as the biggest peak values along the middle line and their change trends.

### 3.2. Wall Shear Stress

**Figure 8:**
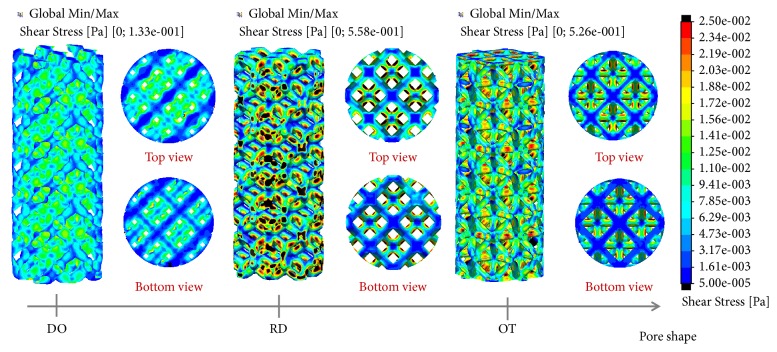
Flow shear stress distributions of the three porous scaffolds.

**Figure 9:**
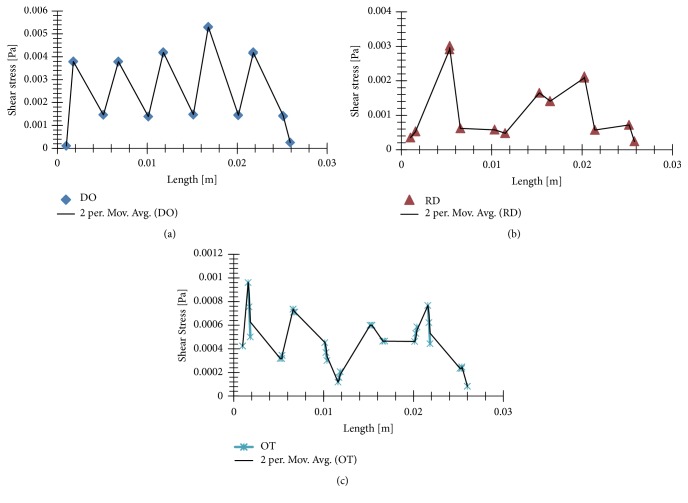
Flow shear stress distributions and change trends along the middle line: (a) DO; (b) RD; (c) OT.

Figure 8 showed the shear stress distributions on the wall surface of the three porous structures. Taking into account previous findings [28–30] the cloud chart were limited to from 0.05 to 25 mPa. The black color marked (>25 mPa or <0.05 mPa) in the figures mean that wall shear stress were not suitable for cell viability and proliferation, and the red color marked usually suggested bigger value than others. Undoubtedly, DO had the least black colors in the three shapes, while RD had the most black colors. However, RD (peak value: 0.558 Pa) and OT (peak value: 0.526 Pa) were seemed to have bigger wall shear stress than DO (peak value: 0.133 Pa). Further on, subtle shear stress distributions and change trends of the wall shear stresses along the middle line were displayed in Figure 9, and significantly difference were depicted vividly.

## 4. Discussions

It is well known that tortuosity (*τ*) is a property of curve and commonly used to describe diffusion in porous media. The mathematical formula of tortuosity is

(4) $$\begin{array}{c} \tau =\frac{L}{L_{0}} \end{array}$$

where *L* and *L*_0_ are the actual length and straight length of fluid channel, respectively. When *L*_0_ keeps same, *τ* would increase with the increasing of *L*. As illustrated in Figure 4, it was evident that flow trajectory (*L*) of DO was more tortuous than OT and RD visually. In this sense, DO should display the biggest tortuosity (*τ*) in the three shapes. According to Xiao [31] and Fan's [32] views, the greater tortuosity is conducive to improving the degree of tissue adhesion and implant fixation when the pore size is enough large, and the channels for fresh tissue and nutrient transport are smoother. Considering that the aperture of DO was the largest (Figure 1(b)) in the three shapes, it could be predicted that DO may have more implant fixation advantages than OT and RD due to the biggest tortuosity.

In addition, flow velocity is another indicator to evaluate the properties of mass transfer [33–35]. Considering descending order of the maximum flow velocities was RD (5.177 mm/s), OT (4.381 mm/s), and DO (3.941 mm/s) in the global view (Figure 4). It was worth noting that RD and OT have better mass transfer performance than DO shapes. Fortunately, inner flow velocities on the diagonal section and middle section (Figure 5) showed completely same global maximum values and same sequence (RD>OT>OT) as Figure 4 portrayed. As a result, abovementioned conclusion should be proved and trusted directly. Along this line of consideration, for DO shape, it could be found that peak velocity on the diagonal section (3.867 mm/s) was closer to the global maximum values (3.941 mm/s) than middle section (3.425 mm/s); namely, diagonal unit cell was likely to bring bigger velocity near to the diagonal section (Figure 6). However, for RD and OT shapes, it was well documented that the discrepancies of global maximum values and peak values on diagonal and middle section were bigger than DO shape (Figure 6). Perhaps, there was no clear rule for RD and OT, but it was worth noting that the maximum values should not appear on the diagonal section and middle section. Further on, as illustrated in Figure 7, different locations of the biggest peak values for flow velocity along the middle line were found. For the DO shape, the biggest peak value was found on the third crest, which was located in the intermediate of the inlet and outlet (Figure 2). However, for RD and OT shapes, their biggest peak values were on the first crest, which were near to the inlet (Figure 2). In the meantime, it is of interest that different change trends of the flow velocities were also found along the middle line in spite of similar waveforms (Figure 7). From the first crest to the fifth crest, RD and OT displayed decreasing peak values gradually, but for DO, the peak value increased firstly and then decreased (Figure 7). It should be noted that pore shape and symmetrical type may be the only causes for these results. Eventually, from the point of flow trajectory and velocity, it could be inferred that DO shape exhibits more advantages on implant fixation than OT and RD, but OT and RD presented better mass transfer performance than DO shape.

Additionally, in this study, by the results of wall shear stress (Figure 8), the least black colors were found on DO shape, and RD and OT displayed more black colors. According to the views of Cartmell [29] and Raimondi [30], it could be concluded that DO has more appropriate adhesion areas than OT and RD, which would provide a better surroundings for cell adhesion and tissue regeneration [36, 37]. Meanwhile, as illustrated in Figure 1(b), DO also had bigger surface area and surface-to volume ratio than DO and OT. Then, above conclusion would be more persuasive and confirmed. Moreover, maximum values of wall shear stress on RD and OT shapes were observed bigger than DO due to more red colors appearing (Figure 8). It could be guessed that DO porous implant may have homogeneous and gentle growth environment for cell proliferation relatively, but for RD and OT, uneven shear stress stimulation would lead to differentiation and asynchrony in different regions of the wall surface. In this regard, the type and growth characteristics of the cell should play a critical role in unit cell selection and tissue regeneration [37].

Further on, change trends of wall shear stresses along the middle line (Figure 9) also had confirmed above difference and relationships in Figure 8. It seemed that DO displayed similar and gentle value along the middle line, and some change trend should be found (Figure 9). However, OT and RD showed unstable values and messy trends. Taking into consideration previous studies [19], unit cell also influenced cell proliferation and activity by affecting the efficiency of shear stimuli to cells under perfused culture, and stress with some frequency had been shown to be favorable for bone regeneration in vitro [38]. Herein, from the point of wall shear stress, it could be concluded that DO shape has some advantages than RD and OT shapes because of more appropriate adhesion areas and regular mechanical stimulation.

As already stated above, although the same porosities (70%), scaffold size (*ϕ*10×25), and simulation environment (DMEM) were shared for DO, RD, and OT, different flow trajectory, velocity, and wall shear stress were found because of unit cell morphology and symmetrical type. Towards this end, the differences between diagonal-symmetrical unit cell and midline-symmetrical unit cell were tentatively confirmed. These findings could provide proofs for the difference between diagonal-symmetrical and midline-symmetrical unit cells and related porous implants. In the meantime, the importance of pore morphology and symmetrical type was also demonstrated [7, 8]. Comprehensively considering the results of flow trajectory, velocity, and wall shear stress, it could be deduced that diagonal-symmetrical shape may have some advantages on implant fixation (bigger tortuosity), cell adhesion (more appropriate adhesion areas), and tissue regeneration (regular mechanical stimulation) compared to midline-symmetrical shape, but midline-symmetrical shape is likely to have super mass transfer performance (bigger flow velocity) compared to diagonal-symmetrical shape. Overall, the results and validity in this study are credible. On the one hand, above findings and inference are not contradict each other, and all the results could be explained and complemented. Meanwhile, as studied before, some findings of this study are consistent with previous literatures [39–41], such as the importance of pore morphology, pore size, surface area, and the advantages of DO shape. On the other hand, in contrast to the conventional cell culture [6] and animal experiment [42], CFD is also an reliable and fast and of low cost method for the flow fluid evaluation for porous implant [37] and regarded as one of the most potential candidates for future patient-specific implants' inspection [43], whose validity was also confirmed by Gómez [20], Olivares [21], Ardiyansyah Syahrom [22], H. Montazerian [19], Chen [28], and so on. Finally, in fluidic optimization, a level-set algorithm for the steady-state Navier–Stokes flow was established, where the solid–fluid interface was determined for maximizing permeability and minimizing energy dissipation in the periodic porous materials [44, 45]. Therefore, the findings in this study are reliable, which could be used to predict biological performance of porous structure with different unit cells.

Eventually, there are also some limitations and future works in this study. Firstly, only three shapes of unit cells with 70% porosity were studied and simulated, which is not enough to meet various needs in practice. In order to create a useful unit cell library, mass transport properties of more unit cells and porosities should be studied in the future. Secondly, cell and animal experiments with the three unit cells were not involved in this study. Future work in vitro and in vivo should be conducted systematically.

## 5. Conclusions

In this study, flow performance of porous structure with diagonal-symmetrical and midline-symmetrical unit cells was evaluated and predicted when the porosities are same. By the results, it can be inferred that porous structures with midline-symmetrical unit cell may have superior mass transport properties than diagonal-symmetrical structure because of bigger flow velocity. However, it seemed that diagonal-symmetrical shape has bigger tortuosity, more appropriate adhesion areas, and more homogeneous and regular shear stimuli than midline-symmetrical shape, which could provide a better environment for implant fixation, cell adhesion, and tissue growth. Additionally, the importance and difference of pore morphology and symmetrical type were also demonstrated. In summary, each unit cell has its own share of merits and demerit;, this study can be utilized to tailor and evaluate the design quantitatively, each with its mass transport properties and tissue regeneration properties.

## Figures and Tables

**Table 1:** Details of CFD simulations.

Unit cell	Scaffolds	Cells	Fluid cells	Solid cells	Partial cells	Iteration	Time*∗* (Min)	Cell volume (mm^3^)
DO	*φ*10×25	411978	251099	56395	104484	264	37	0.46
RD		388431	202221	64009	122201	411	68	0.50
OT		405924	211140	68686	126098	391	46	0.50

*∗Computer configuration*

CPU: Intel(R) Xeon(R) CPU E5-2620 v3 @ 2.40GHz; memory (RAM):32.0GB; OS:64 bits.

## Data Availability

The figure and table data used to support the findings of this study are included within the article.

## References

[1] Hutmacher D. (2000). Scaffolds in tissue engineering bone and cartilage. *Biomaterials*.

[2] Preethi S. S., Haritha M. A., Viji C. S. (2018). Bone tissue engineering: scaffold preparation using chitosan and other biomaterials with different design and fabrication techniques. *International Journal of Biological Macromolecules*.

[3] Karageorgiou V., Kaplan D. (2005). Porosity of 3D biomaterial scaffolds and osteogenesis. *Biomaterials*.

[4] Senatov F., Anisimova N., Kiselevskiy M. (2017). Polyhydroxybutyrate/hydroxyapatite highly porous scaffold for small bone defects replacement in the nonload-bearing parts. *Journal of Bionic Engineering*.

[5] Habib F. N., Nikzad M., Masood S. H., Saifullah A. B. M. (2016). Design and development of scaffolds for tissue engineering using three-dimensional printing for bio-based applications. *3D Printing and Additive Manufacturing*.

[6] Liu Q., Li W., Cao L. (2017). Response of MG63 osteoblast cells to surface modification of Ti-6Al-4V implant alloy by laser interference lithography. *Journal of Bionic Engineering*.

[7] Jin Q.-M., Takita H., Kohgo T. (2000). Effects of geometry of hydroxyapatite as a cell substratum in BMP-induced ectopic bone formation. *Journal of Biomedical Materials Research*.

[8] Kumar A., Biswas K., Basu B. (2015). Hydroxyapatite-titanium bulk composites for bone tissue engineering applications. *Journal of Biomedical Materials Research Part A*.

[9] Wong K., Scheinemann P. (2018). Additive manufactured metallic implants for orthopaedic applications. *Science China Materials*.

[10] Murr L. E., Gaytan S. M., Martinez E., Medina F., Wicker R. B. (2012). Next generation orthopaedic implants by additive manufacturing using electron beam melting. *International Journal of Biomaterials*.

[11] Hooyar A., Shima E. H., Damon K. (2018). Recent developments and opportunities in additive manufacturing of titanium-based matrix composites: a review. *International Journal of Machine Tools and Manufacture*.

[12] Kim J.-K., Lee S.-B., Yang S.-Y. (2018). Cranioplasty using autologous bone versus porous polyethylene versus custom-made titanium mesh: a retrospective review of 108 patients. *Journal of Korean Neurosurgical Society*.

[13] Yang K., Zhou C., Fan H. (2017). Bio-functional design, application and trends in metallic biomaterials. *International Journal of Molecular Sciences*.

[14] Tang D., Tare R. S., Yang L.-Y., Williams D. F., Ou K.-L., Oreffo R. O. C. (2016). Biofabrication of bone tissue: approaches, challenges and translation for bone regeneration. *Biomaterials*.

[15] Zadpoor A. A. (2017). Mechanics of additively manufactured biomaterials. *Journal of the Mechanical Behavior of Biomedical Materials*.

[16] Wang X., Xu S., Zhou S. (2016). Topological design and additive manufacturing of porous metals for bone scaffolds and orthopaedic implants: a review. *Biomaterials*.

[17] Zhang C., Xie S., Li S. (2012). Flow patterns and wall shear stress distribution in human internal carotid arteries: the geometric effect on the risk for stenosis. *Journal of Biomechanics*.

[18] Liu X., Fan Y., Sun A., Deng X. (2013). Numerical simulation of nucleotide transport in the human thoracic aorta. *Journal of Biomechanics*.

[19] Montazerian H., Zhianmanesh M., Davoodi E. (2017). Longitudinal and radial permeability analysis of additively manufactured porous scaffolds: effect of pore shape and porosity. *Materials and Corrosion*.

[20] Gómez S., Vlad M. D., López J., Fernández E. (2016). Design and properties of 3D scaffolds for bone tissue engineering. *Acta Biomaterialia*.

[21] Olivares A. L., Marsal È., Planell J. A., Lacroix D. (2009). Finite element study of scaffold architecture design and culture conditions for tissue engineering. *Biomaterials*.

[22] Syahrom A., Abdul Kadir M. R., Abdullah J., Öchsner A. (2013). Permeability studies of artificial and natural cancellous bone structures. *Medical Engineering & Physics*.

[23] Li J., Chen D., Luan H., Zhang Y., Fan Y. (2018). Numerical evaluation and prediction of porous implant design and flow performance. *BioMed Research International*.

[24] Ahmadi S. M., Hedayati R., Li Y. (2018). Fatigue performance of additively manufactured meta-biomaterials: The effects of topology and material type. *Acta Biomaterialia*.

[25] Li S. J., Xu Q. S., Wang Z. (2014). Influence of cell shape on mechanical properties of Ti-6Al-4V meshes fabricated by electron beam melting method. *Acta Biomaterialia*.

[26] Snelling D., Li Q., Meisel N., Williams C. B., Batra R. C., Druschitz A. P. (2015). Lightweight metal cellular structures fabricated via 3D printing of sand cast molds. *Advanced Engineering Materials*.

[27] Li J., Chen D. S., Luan H. Q. (2017). Mechanical performance of porous implant with different unit cells. *Journal of Mechanics in Medicine & Biology*.

[28] Chen Y., Zhou S., Cadman J., Li Q. (2010). Design of cellular porous biomaterials for wall shear stress criterion. *Biotechnology and Bioengineering*.

[29] Cartmell S. H., Porter B. D., García A. J., Guldberg R. E. (2003). Effects of medium perfusion rate on cell-seeded three-dimensional bone constructs in vitro. *Tissue Engineering Part A*.

[30] Raimondi M. T., Boschetti F., Falcone L. (2002). Mechanobiology of engineered cartilage cultured under a quantified fluid-dynamic environment. *Biomechanics & Modeling in Mechanobiology*.

[31] Xiao D., Yang Y., Su X., Wang D., Sun J. (2013). An integrated approach of topology optimized design and selective laser melting process for titanium implants materials. *Bio-Medical Materials and Engineering*.

[32] Fan J., Jia X., Huang Y., Fu B. M., Fan Y. (2016). Greater scaffold permeability promotes growth of osteoblastic cells in a perfused bioreactor. *Journal of Tissue Engineering and Regenerative Medicine*.

[33] Singh R., Lee P. D., Lindley T. C., Dashwood R. J., Ferrie E., Imwinkelried T. (2009). Characterization of the structure and permeability of titanium foams for spinal fusion devices. *Acta Biomaterialia*.

[34] Porter B., Zauel R., Stockman H., Guldberg R., Fyhrie D. (2005). 3-D computational modeling of media flow through scaffolds in a perfusion bioreactor. *Journal of Biomechanics*.

[35] Zhang Z., Yuan L., Lee P. D., Jones E., Jones J. R. (2014). Modeling of time dependent localized flow shear stress and its impact on cellular growth within additive manufactured titanium implants. *Journal of Biomedical Materials Research Part B: Applied Biomaterials*.

[36] Bacabac R. G., Smit T. H., Cowin S. C. (2005). Dynamic shear stress in parallel-plate flow chambers. *Journal of Biomechanics*.

[37] Dholakia R., Sadasivan C., Fiorella D. J., Woo H. H., Lieber B. B. (2017). Hemodynamics of flow diverters. *Journal of Biomechanical Engineering*.

[38] Jing D., Tong S., Zhai M. (2015). Effect of low-level mechanical vibration on osteogenesis and osseointegration of porous titanium implants in the repair of long bone defects. *Scientific Reports*.

[39] Cavo M., Scaglione S. (2016). Scaffold microstructure effects on functional and mechanical performance: Integration of theoretical and experimental approaches for bone tissue engineering applications. *Materials Science and Engineering C: Materials for Biological Applications*.

[40] Wang Z., Wang C., Li C. (2017). Analysis of factors influencing bone ingrowth into three-dimensional printed porous metal scaffolds: a review. *Journal of Alloys and Compounds*.

[41] Pei X., Zhang B., Fan Y. (2017). Bionic mechanical design of titanium bone tissue implants and 3D printing manufacture. *Materials Letters*.

[42] Van Oirschot B. A. J. A., Eman R. M., Habibovic P. (2016). Osteophilic properties of bone implant surface modifications in a cassette model on a decorticated goat spinal transverse process. *Acta Biomaterialia*.

[43] Lemaire T., Kaiser J., Naili S. (2015). Three-scale multiphysics modeling of transport phenomena within cortical bone. *Mathematical Problems in Engineering*.

[44] Azzam M. I. S., Dullien F. A. L. (1976). Calculation of the permeability of porous media from the navier-stokes equation. *Industrial & Engineering Chemistry Fundamentals*.

[45] Zhou S., Li Q. (2008). A variational level set method for the topology optimization of steady-state Navier-Stokes flow. *Journal of Computational Physics*.

